# Supplementary figures and images for: Urinary microRNAome in healthy cats and cats with pyelonephritis or other urological conditions
Source: PLoS One. 2022 Jul 20;17(7):e0270067. doi: 10.1371/journal.pone.0270067 (PMC9299306; doi:10.1371/journal.pone.0270067)

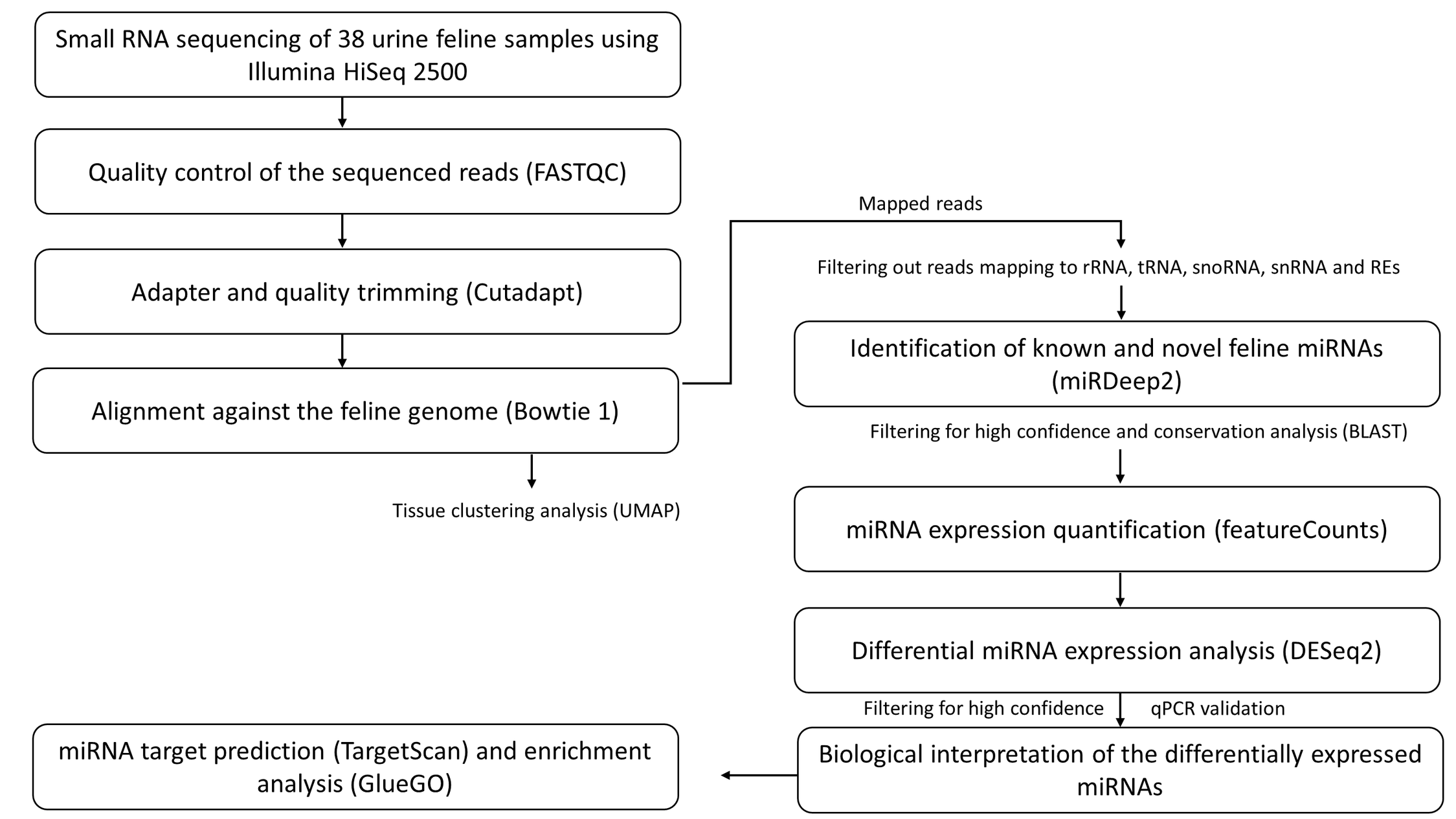

Supplement: S1 Fig — Including the identification of known and putative novel miRNAs, miRNA abundance profiling and differential abundance analysis. rRNA: ribosomal RNA; tRNA: transfer RNA; snoRNA: small nucleolar RNA; snRNA: small nuclear RNA; RE: repeat elements; qPCR: quantitative real-time PCR. (TIF) [file pone.0270067.s001.tif]

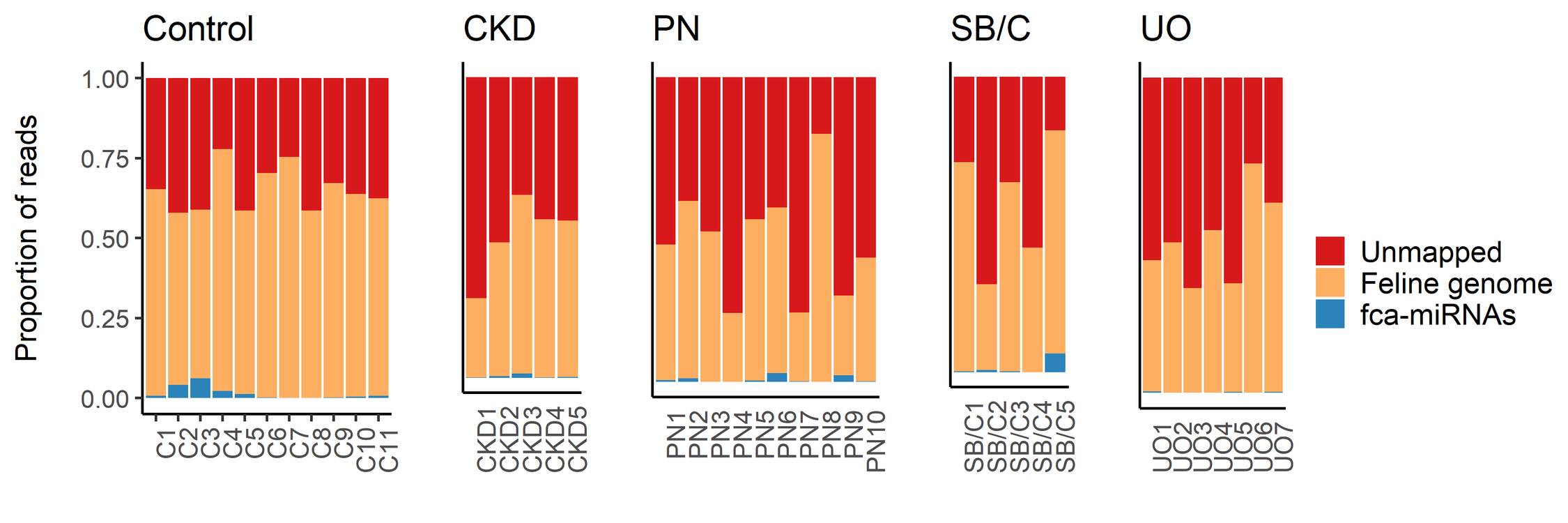

Supplement: S2 Fig — CKD: Chronic kidney disease; PN: Pyelonephritis; SB/C: Subclinical bacteriuria/Cystitis; UO: Ureteral obstruction. (TIF) [file pone.0270067.s002.tif]

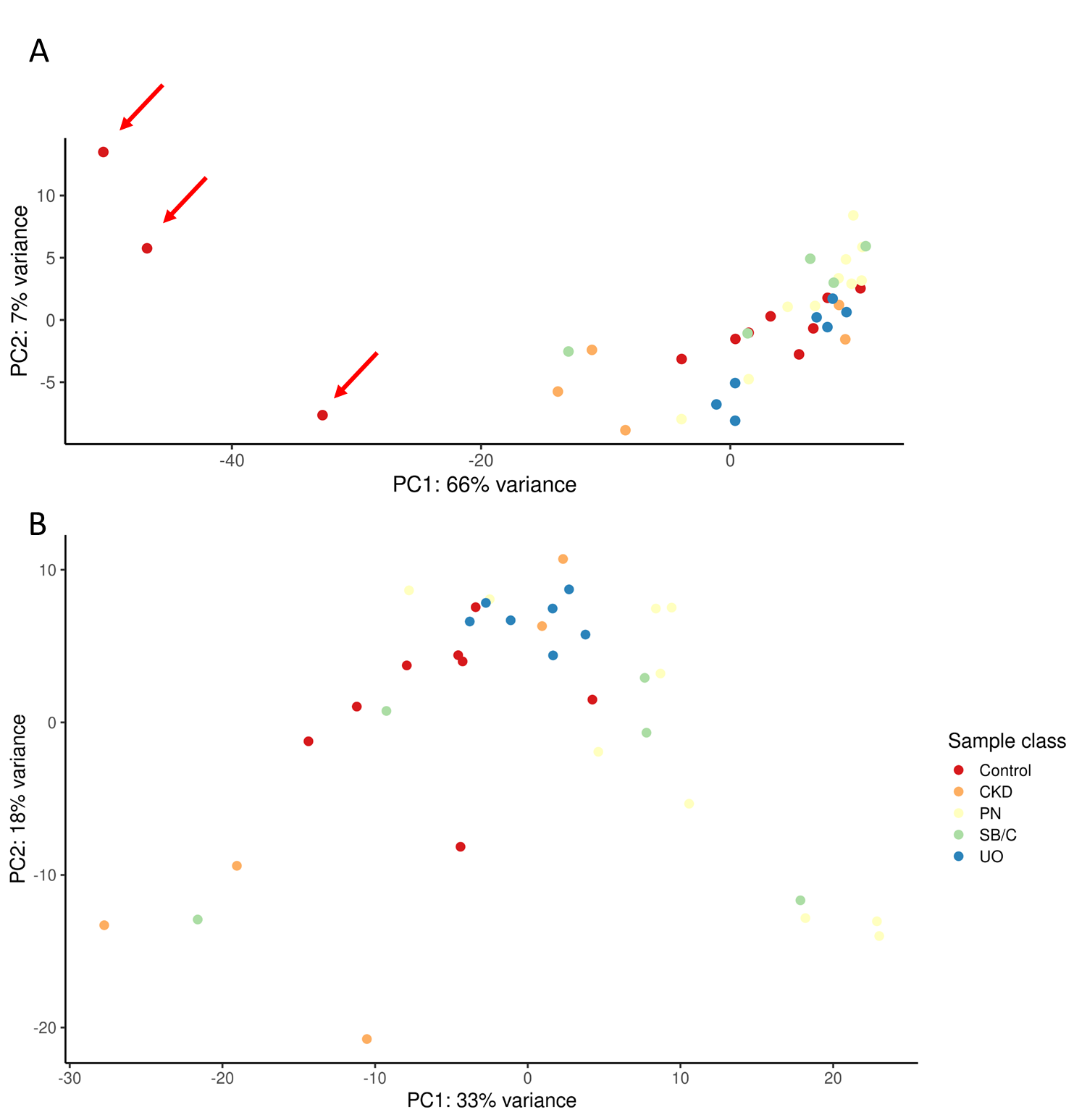

Supplement: S3 Fig — A. PCA of urine samples on the basis of normalized read counts of the known and putative novel miRNAs for the 38 samples initially processed. The red arrows indicate the outlier Control samples (C5, C6 and C7). B. PCA excluding the high outlier samples. CKD: Chronic kidney disease; PN: Pyelonephritis; SB/C: Subclinical bacteriuria/Cystitis; UO: Ureteral obstruction. (TIF) [file pone.0270067.s003.tif]

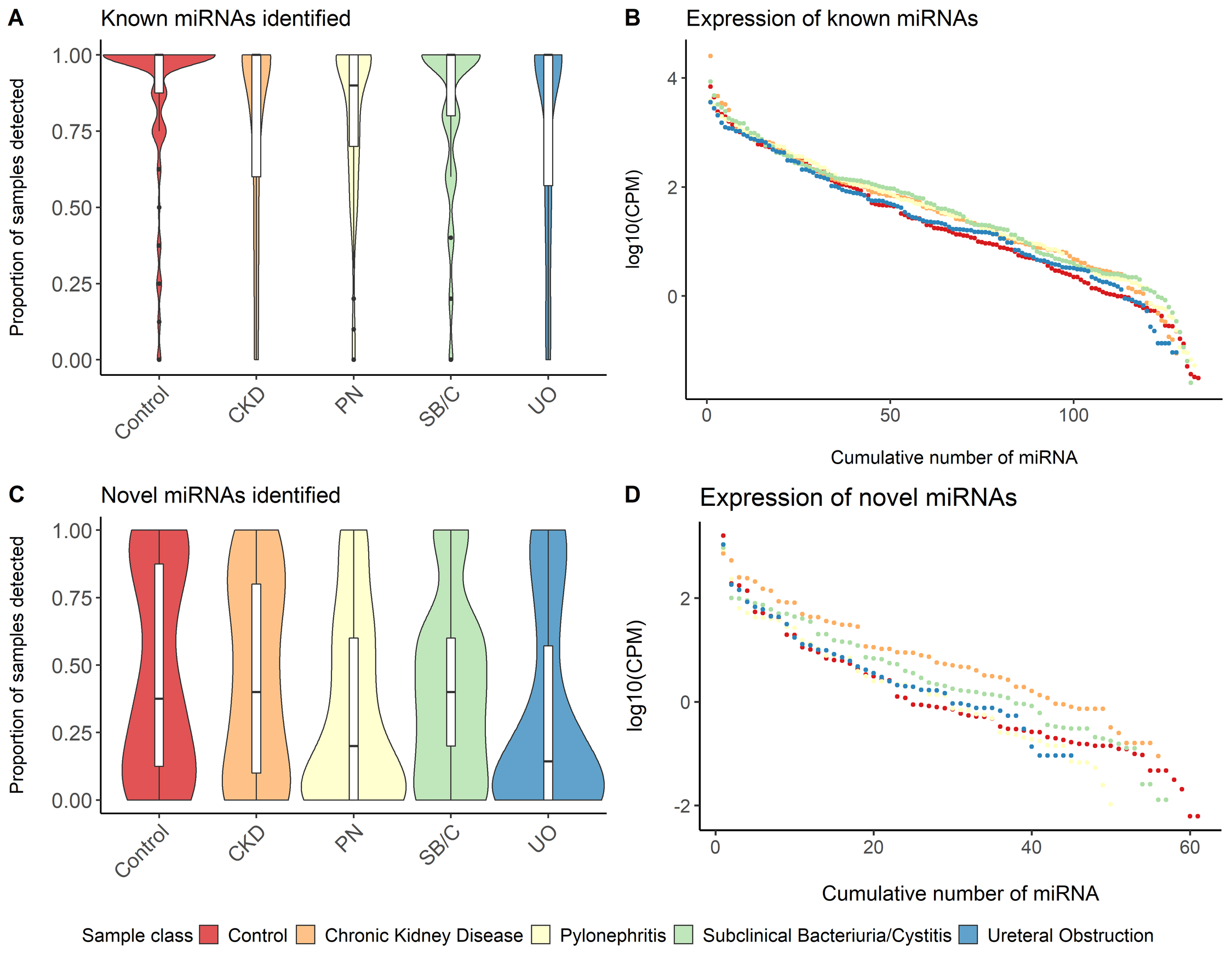

Supplement: S4 Fig — A. Proportion of samples for which each of the known miRNAs across the different groups were detected. B. Cumulative abundance of the known feline miRNAs. The dots indicate the log10 of the miRNA abundance for each miRNA. miRNAs are sorted in each group in a decreasing order by their miRNA abundance on the x-axis, independently for each group. C. Proportion of samples for which each of the putative novel miRNA candidates across the different groups were detected. D. Cumulative abundance of the putative novel miRNAs. The dots indicate the log10 of the miRNA abundance for each miRNA. miRNAs are sorted in each group in a decreasing order by their miRNA abundance on the x-axis, independently for each group. CKD: Chronic kidney disease; PN: Pyelonephritis; SB/C: Subclinical bacteriuria/Cystitis; UO: Ureteral obstruction, CPM: Counts per million. (TIF) [file pone.0270067.s004.tif]

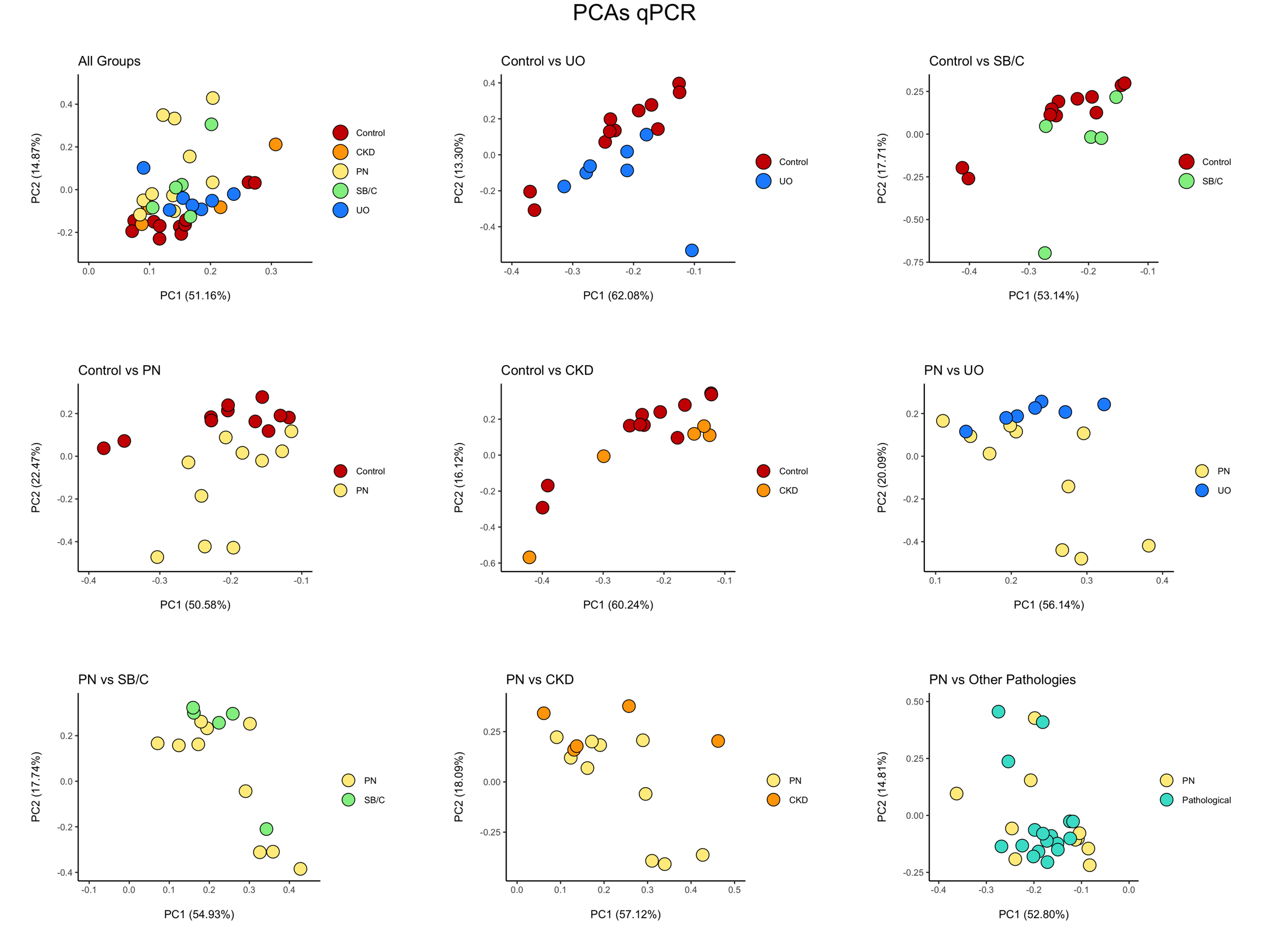

Supplement: S5 Fig — All samples together (all groups), as well as each one of the contrasts considered (Controls vs. PN; Control vs. SB/C; Control vs. UO; Control vs. CKD; PN vs. SB/C; PN vs. UO; PN vs. CKD and PN vs. other Pathologies) are shown. CKD: Chronic kidney disease; PN: Pyelonephritis; SB/C: Subclinical bacteriuria/Cystitis; UO: Ureteral obstruction. (TIF) [file pone.0270067.s005.tif]

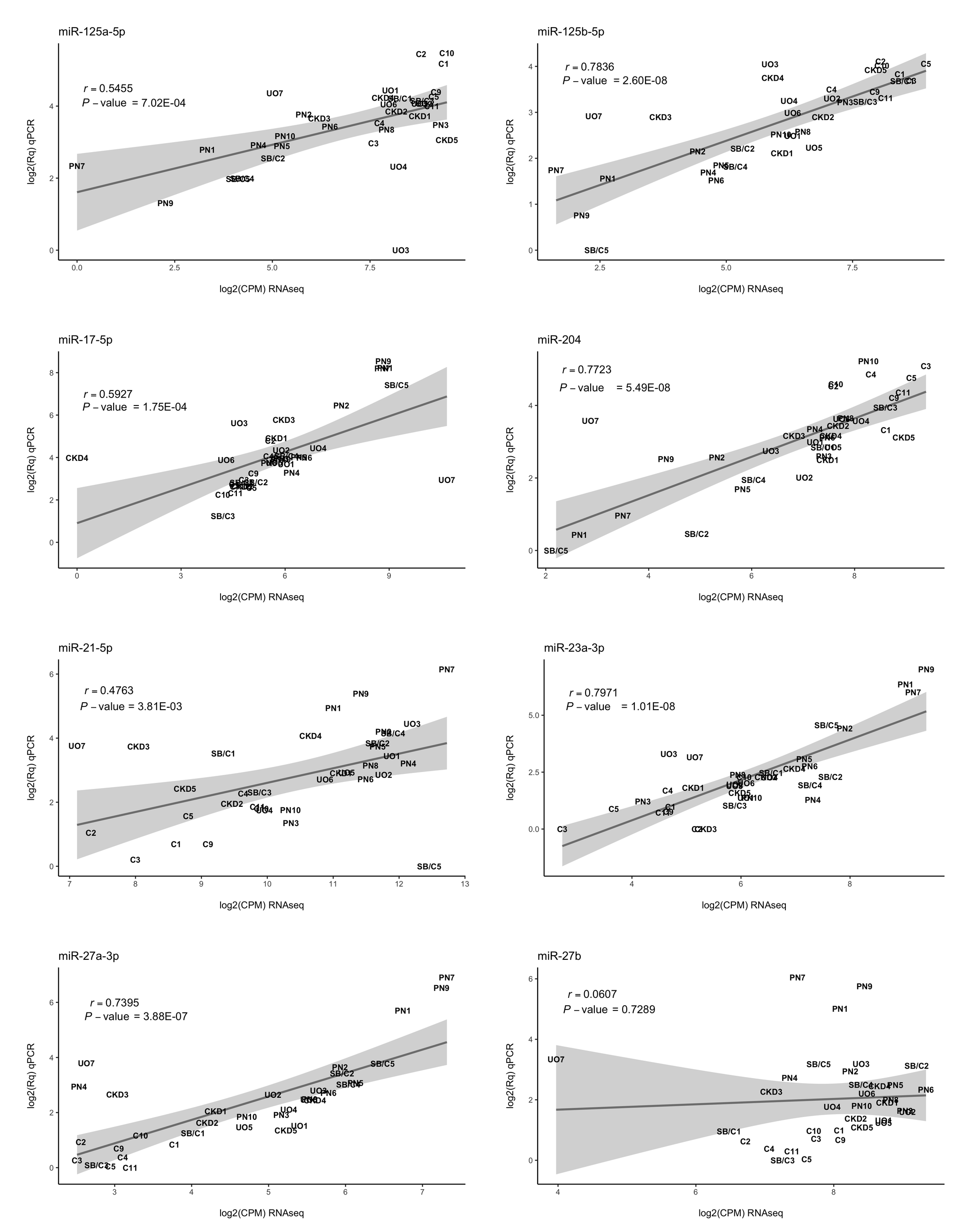

Supplement: S6 Fig — CKD: Chronic kidney disease; PN: Pyelonephritis; SB/C: Subclinical bacteriuria/Cystitis; UO: Ureteral obstruction, CPM: Counts per million, Rq: Relative quantities. (TIF) [file pone.0270067.s006.tif]

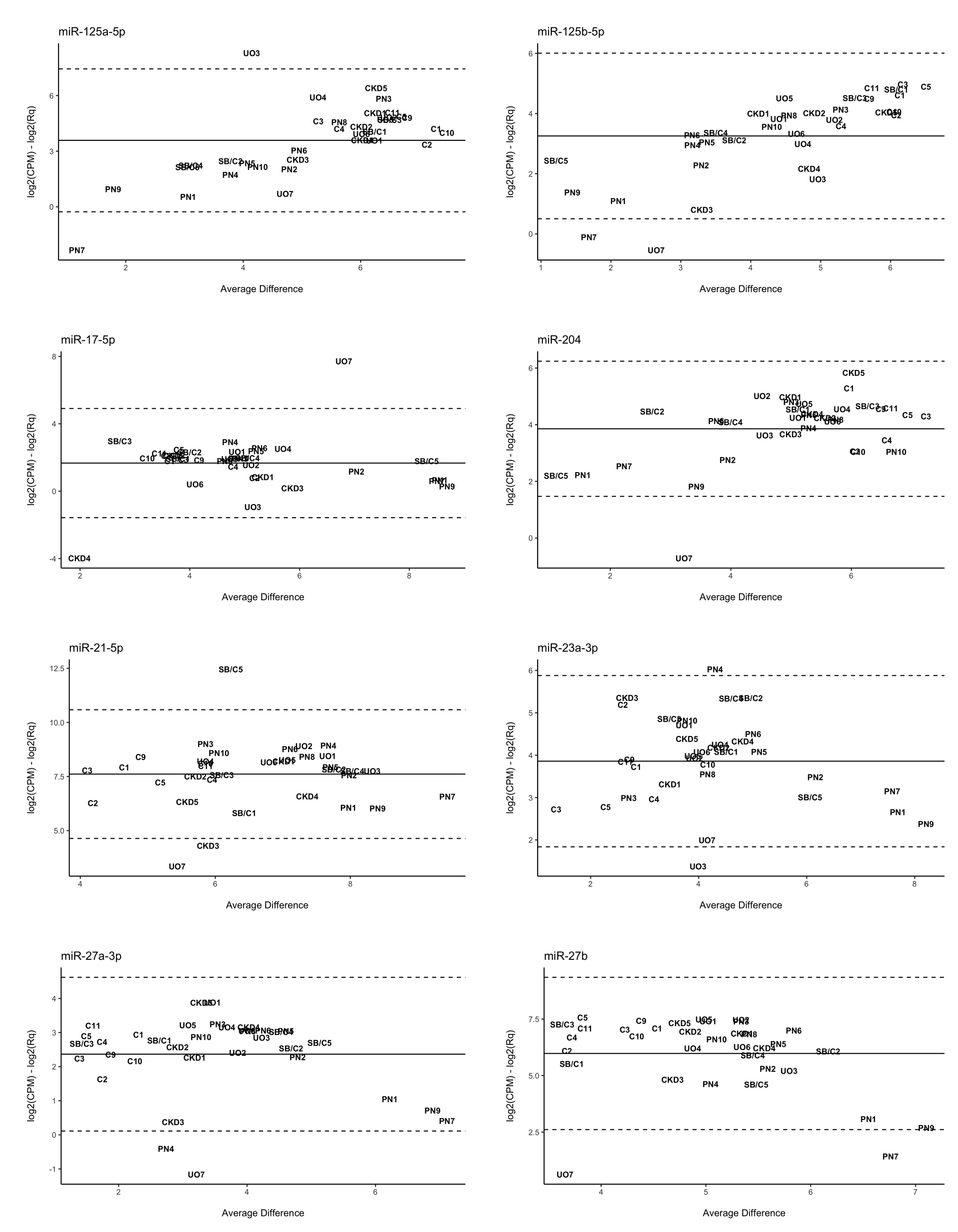

Supplement: S7 Fig — The data presented is from selected miRNAs that were DA (|log2FC| ≥ 1.5 for qPCR and |log2FC| ≥ 2 for small RNAseq; q-value < 0.05) using both methodologies. CKD: Chronic kidney disease; PN: Pyelonephritis; SB/C: Subclinical bacteriuria/Cystitis; UO: Ureteral obstruction, CPM: Counts per million, Rq: Relative quantities. (TIF) [file pone.0270067.s007.tif]
