# Supplementary material for: Urinary microRNAome in healthy cats and cats with pyelonephritis or other urological conditions
Source: PLoS One. 2022 Jul 20;17(7):e0270067. doi: 10.1371/journal.pone.0270067 (PMC9299306; doi:10.1371/journal.pone.0270067)
Supplement: Attachment — Submitted filename: Response to reviewers.docx [file pone.0270067.s018.docx]

**UNIVERSITY OF COPENHAGEN**

**Department of Veterinary and**

**Animal Sciences**

Dear Academic Editor Silvia Sabattini,	16^th^ May, 2022

The authors thank the reviewers and the editor for their effort in reviewing this manuscript.

We have used the word “sex” (instead of gender) in the whole manuscript. After checking the whole manuscript, I can confirm that the word “gender” was only used once in the response to reviewers letter. So I have submitted again the final version of the manuscript (clean and with track changes) which is identical to the version of 1^st^ April.

On behalf of the authors,

Sincerely,

Susanna Cirera
